# Effects of light deprivation on visual evoked potentials in migraine without aura

**DOI:** 10.1186/1471-2377-11-91

**Published:** 2011-07-27

**Authors:** Gianluca Coppola, Julien Crémers, Pascale Gérard, Francesco Pierelli, Jean Schoenen

**Affiliations:** 1 G.B. Bietti Eye Foundation-IRCCS, Dept of Neurophysiology of Vision and Neuroophtalmology, Rome, Italy; 2 Headache Research Unit. University Dept. of Neurology, Liège University, Belgium; 3 Dept of Medico-Surgical Sciences and Biotechnologies, "Sapienza" University of Rome Polo Pontino, Latina, Italy; 4 IRCCS-Neuromed, Pozzilli (IS), Italy; 5 GIGA-Neurosciences, Liège University, Liège, Belgium

## Abstract

**Background:**

The mechanisms underlying the interictal habituation deficit of cortical visual evoked potentials (VEP) in migraine are not well understood. Abnormal long-term functional plasticity of the visual cortex may play a role and it can be assessed experimentally by light deprivation (LD).

**Methods:**

We have compared the effects of LD on VEP in migraine patients without aura between attacks (MO, n = 17) and in healthy volunteers (HV, n = 17). Six sequential blocks of 100 averaged VEP at 3.1 Hz were recorded before and after 1 hour of LD. We measured VEP P100 amplitude of the 1^st ^block of 100 sweeps and its change over 5 sequential blocks of 100 responses.

**Results:**

In HV, the consequence of LD was a reduction of 1^st ^block VEP amplitude and of the normal habituation pattern. By contrast, in MO patients, the interictal habituation deficit was not significantly modified, although 1^st ^block VEP amplitude, already lower than in HV before LD, further decreased after LD.

**Conclusions:**

Light deprivation is thought to decrease both excitatory and subsequent inhibitory processes in visual cortex, which is in line with our findings in healthy volunteers. The VEP results in migraine patients suggest that early excitation was adequately suppressed, but not the inhibitory mechanisms occurring during long term stimulation and habituation. Accordingly, deficient intracortical inhibition is unlikely to be a primary factor in migraine pathophysiology and the habituation deficit.

## Background

Deficient habituation of pattern reversal visual evoked potentials (VEPs) during long lasting stimulus repetition characterizes migraine patients during the pain-free interval [1-3].

This abnormal information processing could be attributed to hypofunctioning intracortical inhibitory circuits [4,5] or to a reduced preactivation level of the visual cortex, which may be due to hypoactive aminergic projections from the brainstem [1,2].

Immediate and longer-lasting cortical changes, such as those induced by repetitive sensory stimuli, are thought to reflect central nervous system (CNS) plasticity and modifications of synaptic effectiveness in the stimulated cortex through short- and long- term depression (LTD) [6,7]. We suggested therefore that altered functional plasticity of sensory cortices could be responsible for the electrophysiological abnormalities found in migraine [8].

Light deprivation (LD) is a well-known, validated in vivo model for the study of functional plasticity in the visual system [9,10]. LD reduces time dependently peripheral [11,12], and central [13,14] neural activity in animal models, which is accompanied by behavioural changes [14]. In humans, LD slows the electroencephalogram (EEG), by increasing low-band power and decreasing alpha activity [15,16].

In studies of visually deprived animals [17-19] and humans [20,21], smaller VEP amplitudes have been observed. Conversely, phosphene thresholds after transcranial magnetic stimulation (TMS) were decreased after 1 hour of light deprivation [22,23] which was attributed to decreased excitability of cortical inhibitory interneurons [22].

We decided therefore to use light deprivation in order to investigate more precisely long-term functional plasticity of the visual cortex and the role of cortical inhibition in migraine patients between attacks in whom habituation is absent and in healthy subjects who have a normal habituation. We measured VEP amplitudes to low numbers of stimuli and VEP habituation over sequential blocks during uninterrupted durable visual stimulation.

## Methods

Subjects - We enrolled 17 consecutive migraine patients without aura (MO, ICHD-II code 1.1) (14 women and 3 men, mean age 28.9 ± 12.1 years) from our headache clinic. They underwent VEP recordings during the interictal period, i.e. at least three days before and after the recordings. No preventive anti-migraine drugs were allowed during the preceding 3 months. For comparison, 17 healthy subjects of comparable age and gender distribution (14 women and 3 men, mean age 28.8 ± 11.4 years) were recruited among medical school students and healthcare professionals. They had to be devoid of any overt medical condition, personal or family history of migraine or epilepsy, and regular drug intake. To minimize variability due to hormonal influences on cortical excitability, female subjects were always recorded at mid-cycle.

Participants taking medications on a regular basis and subjects who failed to reach a best corrected visual acuity of > 8/10 were excluded. None of the enrolled subjects had sleep deprivation or alcohol ingestion the day preceding the recordings. Caffeinated beverages were not allowed on the day of recordings. All participants received a complete description of the study and granted written informed consent. The project was approved by the ethical review board of the Faculty of Medicine, University of Liège, Belgium.

Visual evoked potential recordings - Subjects were seated in an acoustically isolated room with dimmed light in front of a TV monitor surrounded by a uniform luminance field of 5 cd/m^2^. To obtain a stable pupillary diameter, each subject adapted to the ambient room light for 10 min before the VEP recording. VEPs were elicited by monocular stimulation. Visual stimuli consisted of full-field checkerboard patterns (contrast 80%, mean luminance 250 cd/m^2^) generated on a TV monitor and reversed in contrast at a rate of 3.1/s. At the viewing distance of 80 cm, the single check edges subtended a visual angle of 15 minutes. Subjects were instructed to fixate a red dot in the middle of the screen with the left eye covered by a patch to maintain stable fixation. VEPs were recorded from the scalp through pin electrodes positioned at Oz (active electrode) and Fz (reference electrode, 10/20 system). A ground electrode was placed on the right forearm. The evoked potential signals were amplified by CED™ 1902 preamplifiers (band-pass 0.05-2000 Hz, Gain 1000) and recorded with a CED™ 1401 device (Cambridge Electronic Design Ltd, Cambridge, UK). A total of 600 consecutive sweeps each lasting 200 ms were collected and sampled at 4000 Hz.

After applying off-line a 45 Hz low-pass digital filter, cortical responses were partitioned in 6 sequential blocks of 100, consisting of at least 95 artefact-free sweeps. Responses in each block were averaged off-line ("block averages") using the Signal™ software package version 3.10 (CED Ltd).

**Figure 1:**
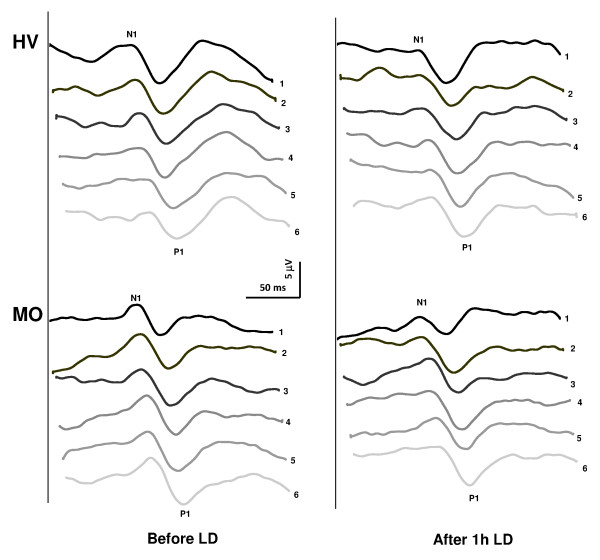
**Representative recordings of visual evoked potential (VEP): habituation at baseline (left) and after 1 h light deprivation (right) in a healthy subject [HS] and a migraine without aura patient [MO]**.

VEP were off-line analysed by one investigator (J.C.) not blinded for subjects' diagnosis. VEP components were identified according to their latencies: N1 was defined as the most negative peak between 60 and 90 ms, P1 as the most positive peak following N1 between 80 and 120 ms, and N2 as the most negative peak following P1 at between 125 and 150 ms (Figure 1). We measured peak-to-peak amplitude of the N1-P1 complex. Habituation was defined both as the change in N1-P1 amplitude between the 1^st ^and the 6^th ^block of averages and the slope of the linear regression line over the 6 blocks. VEP habituation was evaluated before and immediately after light deprivation. All recordings were collected in the morning (between 09.00 and 11.00 a.m.) by the same investigator.

Light deprivation (LD) - Light deprivation was obtained by having subjects wear opaque goggles covered by a mask. They had to report complete absence of light perception and freedom of eyelid movement. Subjects were instructed to relax, keep their eyes open and blink as usual. VEPs were recorded before and immediately after 1 hour of LD.

Statistical analyses - We used the Statistical Package for the Social Sciences (SPSS) for Windows, version 15.0 for all analyses. We constructed a multivariate analysis of variance (ANOVA) taking as a within-subject factor "block" and as between-subject factors "Group" (HV, MO) and "session" (before and after LD). A regression analysis was used to disclose linear trends in VEP amplitude across blocks (slope) in each condition and group. The paired Student's t-test was used to compare VEP amplitude in block 1 before and after LD in both groups of subjects. Fisher's least significant difference (LSD) test was used for post hoc analysis. Pearson's correlation test was used to search for correlations among the VEP amplitude slopes and clinical variables. P values smaller than 0.05 were considered to indicate statistical significance.

## Results

VEP recordings from all participants yielded analysable data. Examples of VEP recordings before and after 1 h of light deprivation, obtained from a healthy subject and from a MO patient, are shown in Figure 1.

**Figure 2:**
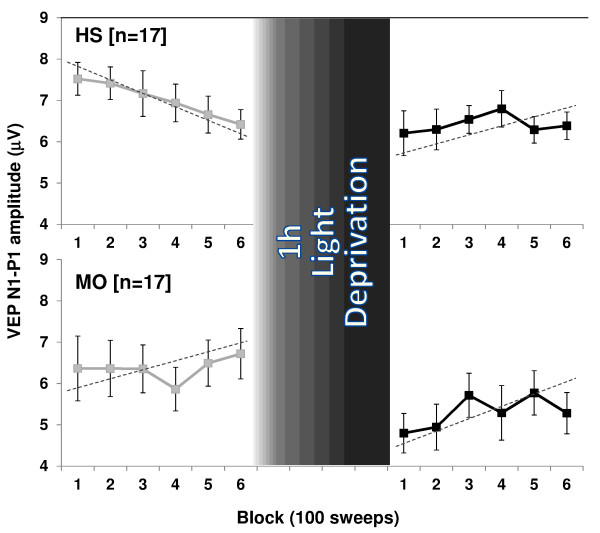
**VEP N1-P1 block amplitudes (mean + SEM) before and after 1 h of light deprivation (LD) in healthy volunteers [HV] and migraine patients without aura [MO]**.

ANOVA for amplitude in averaged VEP blocks disclosed a significant two-way interaction of group by block (F_(5,320) _= 3.72, p = 0.002), and time by block (F_(5,320) _= 3.57, p = 0.003), but not of group by block and time (F_(5,320) _= 1.13, p = 0.340). Linear regression analysis of VEP amplitudes recorded over all 6 blocks differed between sessions in healthy subjects (F_(1,32) _= 10.04, p = 0.003) but not in patients (F_(1,32) _= 0.52, p = 0.47). Post hoc analysis showed that before LD the slope of VEP amplitudes from block 1 to block 6 was negative (-0.22 ± 0.16) in healthy subjects whereas in patients it was positive (+0.04 ± 0.39, Figure 2). Conversely, after 1 hour of LD, the slope of VEP amplitudes from block 1 to block 6 became positive in healthy volunteers (+0.03 ± 0.07), while there was little change in patients (+0.12 ± 0.22).

**Figure 3:**
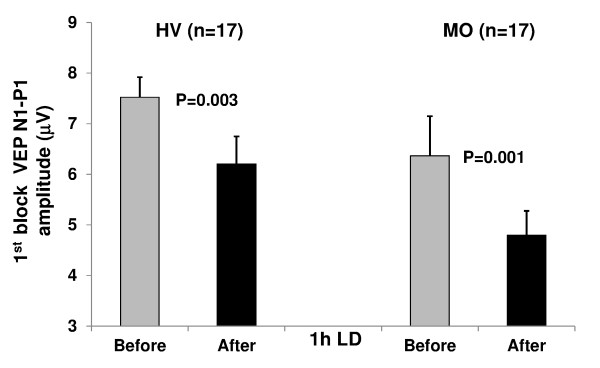
**VEP N1-P1 amplitude (mean + SEM) in the 1^st ^block of averaged responses before and after 1 h of light deprivation (LD) in healthy volunteers [HV] and migraine without aura patients [MO]**.

VEP block 1 amplitude decreased significantly after LD in both healthy subjects (t_(1,17) _= 3.47, p = 0.003) and patients (t_(1,17) _= 4.25, p = 0.001; Figure 3).

With Pearson's test there was no significant correlation between clinical characteristics and VEP amplitude slopes (before and after LD) in migraine patients.

## Discussion

Light deprivation produced differential changes of pattern reversal VEPs in healthy subjects and migraine without aura patients recorded between attacks. Whereas in healthy subjects LD decreased VEP amplitude in block 1 and abolished the normal VEP habituation, in patients it also further decreased the already low VEP amplitude in block 1, but did not significantly change the abnormal VEP habituation pattern.

The reduction in 1^st ^block VEP amplitude after LD is in line with current neurobiological knowledge on LD-induced changes in the visual system. Chronic light deprivation reduces retinal ganglion cell responses [11], induces plastic changes in lateral geniculate nucleus [13], alters neural activity in monoamine brain systems, especially in locus coeruleus, and induces loss of cortical noradrenergic fibres to the visual cortex [14]. These LD-induced changes produce behavioural deficits in decision making and attention tasks in animal models [14]. In humans, prolonged [15] as well as brief visual deprivation for 1 or 2 hours slows the electroencephalogram (EEG) and decreases alpha frequency power [15,16,24]. The latter observation is of particular interest for the LD-induced reduction of VEP amplitudes in the 1^st ^block. It is well known indeed that EEG alpha activity predominates in occipital areas and that it is directly correlated with VEP amplitude in healthy subjects [25]. The changes in alpha power may reflect a change in general arousal [26] and are probably induced by a diffuse activation from the brainstem reticular formation [27,28]. The latter is thought to modulate visual evoked responses [29-31] and, consequently, the mechanisms of LD-induced cortical plasticity [14]. In line with this concept, VEP amplitudes were found reduced in animals during and immediately after LD [17,19,21], which was attributed to an increase of synchronic thalamic inhibitory influences on the cortex, resulting, as in our study, in a decrease of excitatory drive and thus of VEP amplitude [19,21].

The second distinctive finding in our study is that in healthy subjects experimental LD attenuates VEP habituation. In animals it was shown previously that LD reduces excitation at early, but also inhibitory processes at later time points, which results in a loss of VEP habituation during continuous stimulus repetition [19,21]. The dynamics of the excitatory-inhibitory balance that finally results in a lack of long-term depression, i.e. lack of habituation, after 1 h of LD, is likely to depend on glutamatergic and GABAergic mechanisms. In animal models, LD alters NMDA-receptor-dependent synaptic plasticity [13,32,33] and impairs both short- and long- term depression in visual cortex [34]. In animals reared in the dark from birth the effectiveness of inhibitory synapses on the soma of pyramidal neurons in the superficial layers of the visual cortex is markedly reduced [35]. Altered GABA neurotransmission in the visual cortex by LD was also considered to be the mechanism underlying the decrease in magnetophosphene thresholds after LD in healthy humans [22,23]. However, the magnetophosphene and VEP studies are not comparable without reservation, as the habituation phenomenon is only studied in the latter. VEP amplitudes tend already to habituate between the 4^th ^and 6^th ^block of averagings, i.e. during the 4^th ^minute of stimulation (-3.0%, Figure 2), while the decrease of magnetophosphene thresholds is still observed 180 minutes after the end of LD. As suggested by pharmacological studies [36], besides cortical GABAergic inhibition, it is thus likely that excitatory neurotransmitters like acetylcholine and glutamate also play a role in LD-induced visual cortex plasticity.

Another finding in our study is that 1 h LD has no effect on the interictal habituation deficit in migraineurs. This contrasts with a "normal" behavior of the 1^st ^block VEP which, as in healthy volunteers, decreases after LD despite its already lowered amplitude. In migraineurs, unlike healthy subjects, VEP amplitude does not habituate between the 4^th ^and 6^th ^blocks, but on the contrary continues to increase (+6.7%) confirming once more that the brain mechanisms responsible for long-term habituation are malfunctioning in migraine [1-3]. We have shown previously that thalamo-cortical activation is reduced in migraine between attacks [37,38] which might be attributed to functional disconnection of the thalamus from its control by aminergic brainstem nuclei [39]. Light deprivation precisely has an effect on brainstem noradrenergic and serotoninergic neurons in rat [14]. The transient dysfunction of these neurons might worsen the already reduced thalamo-cortical activity, producing an additional decrease in cortical excitation and further reduction of VEP amplitude in the 1^st ^block of averaged responses. In the thalamo-cortical dysrhythmia [40,41] model of migraine, the deficient habituation in later blocks of VEP averagings can be attributed to inefficient lateral inhibition in the visual cortex [37]. It was hypothesized that the deficient habituation in migraineurs is due to a hypofunction of inhibitory cortical interneurons [4,5]. In this case, however, one would expect that after LD that inhibits inhibitory interneurons (see above), the habituation deficit worsens. The fact that this was not our finding does not favor this hypothesis, unless one assumes that the habituation deficit is already maximal before LD in migraine patients and cannot be further increased. It is known that the effect of LD on the visual cortex depends on the underlying level of cortical activation that is not fixed [42] and might be genetically determined [34,42,43]. Taken together, these data suggest that the migraine disease-related decrease in thalamocortical preactivation prevents the occurrence of the normal plastic changes induced by 1 h LD.

## Conclusions

In conclusion, the reduction in VEP 1^st ^block amplitude after LD in healthy subjects and migraineurs might reflect a transient thalamo-cortical dysfunction due to interference with neural activity in brainstem modulatory aminergic centres [14,19,24,44]. This initial inhibitory effect on VEP amplitude is worsened in migraine, probably because of further impairment of an already reduced thalamo-cortical drive. This reduction results in abnormal modulation of the visual cortex and failure of long term VEP inhibitory mechanisms, which prevents further changes in the pre-existing habituation deficit. Consequently, reduced cortical preactivation, and not primary dysfunction of intracortical inhibition, is likely to be the main cause of the habituation deficit found in migraine patients between attacks.

## Competing interests

The authors declare that they have no competing interests.

## Authors' contributions

GC made substantial contributions to analysis and interpretation of data as well as in drafting the manuscript. JC, PG & FP were implied in recording data and analysis. JS was implied in the interpretation of data as well as in drafting the manuscript; gave critical revision of the manuscript for important intellectual content. All authors read and approved the final manuscript.

## Pre-publication history

The pre-publication history for this paper can be accessed here:

http://www.biomedcentral.com/1471-2377/11/91/prepub
